# Health-related quality of life and mortality in patients with pulmonary embolism: a prospective cohort study in seven European countries

**DOI:** 10.1007/s11136-019-02175-z

**Published:** 2019-04-04

**Authors:** Ling-Hsiang Chuang, Pearl Gumbs, Ben van Hout, Giancarlo Agnelli, Sonja Kroep, Manuel Monreal, Rupert Bauersachs, Stephen N. Willich, Anselm Gitt, Patrick Mismetti, Alexander Cohen, David Jimenez

**Affiliations:** 1 Pharmerit International, Rotterdam, The Netherlands; 2 Daiichi-Sankyo Europe GmbH, Munich, Germany; 3 University of Sheffield, Sheffield, UK; 4 University of Perugia, Perugia, Italy; 5 Hospital Universitari Germans Trias I Pujol, Barcelona, Spain; 6 Klinikum Darmstadt, Darmstadt, Germany; 7 Charité - Universitätsmedizin Berlin, Berlin, Germany; 8 Herzzentrum Ludwigshafen, Ludwigshafen, Germany; 9 CHU Saint-Etienne, Hospital Nord, Saint Etienne Cedex 2, France; 10 Guy’s and St Thomas’ NHS Foundation Trust, London, UK; 11 Ramon y Cajal Hospital IRYCIS and Alcala de Henares University, Madrid, Spain

**Keywords:** Venous thromboembolism, Burden of illness, Mortality, Health-related quality of life, Disease registry

## Abstract

**Purpose:**

Little is known about the quality of life following pulmonary embolism (PE). The aim of the study was to assess the 12-month illness burden in terms of health-related quality of life (HrQoL) and mortality, in relation to differences in patient characteristics.

**Methods:**

The PREFER in VTE registry, a prospective, observational study conducted in seven European countries, was used. Within 2 weeks following an acute symptomatic PE, patients were recruited and followed up for 12 months. Associations between patient characteristics and HrQoL (EQ-5D-5L) and mortality were examined using a regression approach.

**Results:**

Among 1399 PE patients, the EQ-5D-5L index score at baseline was 0.712 (SD 0.265), which among survivors gradually improved to 0.835 (0.212) at 12 months. For those patients with and without active cancer, the average index score at baseline was 0.658 (0.275) and 0.717 (0.264), respectively. Age and previous stroke were significant factors for predicting index scores in those with/without active cancer. Bleeding events but not recurrences had a noticeable impact on the HrQoL of patients without active cancer. The 12-month mortality rate post-acute period was 8.1%, ranging from 1.4% in Germany, Switzerland, and Austria to 16.8% in Italy. Mortality differed between patients with active cancer and those without (42.7% vs. 4.7%).

**Conclusion:**

PE is associated with a substantial decrease in HrQoL at baseline which normalizes following treatment. PE is associated with a high mortality rate especially in patients with cancer, with significant country variation. Bleeding events, in particular, impact the burden of PE.

## Introduction

Pulmonary embolism (PE) is a relatively common emergency [1, 2]. PE may be unprovoked or as a complication of underlying diseases such as cancer, medical conditions, and surgical procedures. Several empirical estimates of PE incidence rate from European cohort studies have been reported, ranging from 0.19 to 0.6 per 1000 population per year [3–8]. In contrast, an incidence-based epidemiological model estimated that the PE incidence rate is 0.95 per 1000 population per year in the European Union [9].

PE is, together with deep vein thrombosis (DVT), one of two clinical presentations of venous thromboembolism (VTE) [2]. Long-term morbidity is related to an increased risk of developing recurrent VTE, bleeding, and post-VTE complications including chronic thromboembolic pulmonary hypertension (CTEPH) syndrome and post-thrombotic syndrome (PTS) [9]. In comparison to DVT which has been better studied with respect to health-related quality of life (HrQoL), PE has been under investigated [10]. Some studies have reported the impaired HrQoL of PE patients compared to that of the normal population [10–12]. A recent study in Norway showed that patients with a history of PE have statistically significant lower EQ-5D-3L index score than that of the age/gender-matched control group [10]. So far no study has reported HrQOL of PE patients using the 5-level EQ-5D instrument (EQ-5D-5L) outside of clinical trials.

PE is associated with increased mortality, with the risk being highest soon after diagnosis [13]. It is estimated using a modified incidence-based model that 543,454 VTE-related deaths occurred in the European Union in 2004 (population 454.4 million) [9], whereas a large European registry reported 30-day all-cause PE mortality rate as 5.9% in patients that survived to have the diagnosis confirmed.

In addition, PE is associated with additional resource utilization and indirect costs due to days missed from work and work loss [14–17]. Thus, as a whole, PE causes a significant economic burden to society.

This study aimed to contribute to the current knowledge on the HrQoL of patients with PE in Europe using the results from the PREFER in VTE registry. At the time of the study, a significant number of patients without active cancer received non-VKA oral anticoagulant (NOAC) therapy in place of conventional therapy with vitamin K antagonist (VKA). The primary outcome of the study was HrQoL as measured by the EQ-5D-5L, and a secondary aim was to assess 12-month mortality. Attention was given to differences per country and differences between patients with and without active cancer. The association between patient characteristics, decreased HrQoL, and increased mortality was also investigated. A separate publication is available dealing with healthcare resource use and productivity loss in patients with PE [15].

## Methods

### Setting and study population

The PREFER in VTE registry was a prospective, observational, multicenter study with a follow-up of 12 months which enrolled 3545 consecutive patients from 311 centers in seven European countries: Austria, France, Germany, Italy, Spain, Switzerland, and the UK between January 2013 and July 2014. Prior to study commencement, the registry protocol was approved by the responsible ethics committees for the participating countries and the relevant hospital-based institutional review boards. All patients enrolled in the registry first provided written informed consent. The outline has been described elsewhere [18, 19].

Patients were eligible if they were at least 18 years old, and had a symptomatic, objectively confirmed first time or recurrent acute VTE (the index event) defined as either distal or proximal deep vein thrombosis, pulmonary embolism, or both. Patients were recruited within 2 weeks of the occurrence of the index event and no exclusion criteria applied. Patients were usually recruited post the acute phase of therapy which usually lasts 5–14 days. At baseline, patients were assessed face to face to ascertain demography, previous clinical events, risk factors, comorbidities, PE/DVT symptoms, and previous VTE treatment. At 1, 3, 6, and 12 months, information regarding clinical events (such as VTE events [recurrence] or bleeds occurring during follow-up), treatment, resource utilization, health-related quality of life, and treatment satisfaction during each follow-up interval was collected through telephone calls. The current study concerned PE patients only. PE patients were defined as patients that had either a PE with DVT or a PE without DVT. A total of 1399 PE patients were recruited in the registry.

### Data quality control

The validity of the data was assured by training on data collection ensuring a uniformity and random audits were performed. During these visits, the monitor verified informed consent documentation, source data against medical records, and consecutiveness of enrolment. Hospitals and specialized outpatient centers were included. As hospital-based investigators do not always follow patients for routine care, patients were also asked to participate in follow-ups by phone, safeguarding the collection of resource consumption data. Information was collected directly from the patients during standardized phone calls at 1, 3, 6, and 12 months. Data were checked electronically for completeness and plausibility at the time of entry and additional validation was performed.

### Outcomes

Health-related quality of life was assessed by EQ-5D-5L [20]. EQ-5D-5L has 5 dimensions: mobility, self-care, usual activities, pain/discomfort, and anxiety/depression. Within each dimension, one may have no problem, slight problems, moderate problems, severe problems, or extreme problems (5 levels) [20]. Based on the individual response to the descriptive system (5 dimensions and their responses), a health state can be defined (e.g., 11111 or 21112). For each health state, an index score can be assigned, which represents social preference towards each state. The primary outcome of interest was EQ-5D-5L scores, including the distribution and EQ-5D-5L index score. The secondary outcomes were 12-month mortality rates. Following enrolment in the registry, mortality data were collected at baseline and at each follow-up (1, 3, 6, and 12 months) through a standardized questionnaire filled out by the investigator.

#### Health-related quality of life

EQ-5D-5L scores, both the distribution of responses of each dimension and EQ-5D-5L index score, were presented at baseline and each follow-up point by country and cancer subgroups. The population reference values of EQ-5D-5L index scores, based on the UK EQ-5D-5L valuation study [21], were used to compare with those of the PE population. The extent to which the EQ-5D index score was affected by baseline characteristics (age, gender, BMI, previous clinical events, clinical factors [excluding provoked], comorbidities [excluding cardiovascular disease], and risk factors) plus follow-up events (VTE events [recurrence] or bleeds occurring during follow-up) was assessed. The choice was made to exclude symptoms at baseline from the analysis, as the inclusion of PE symptoms leads to bias in the coefficients of the other baseline characteristics, impairing the interpretation of the impact of these variables on health-related quality of life. Moreover, PE symptoms and health-related quality of life are highly correlated. Treatment was not included in the analyses as this was highly correlated with country, as NOACs were not being reimbursed in Italy and Spain at the time of the data collection. Tobit regression with repeated measures was used to capture the fact that there is an upper limit of one and multiple data points from the same patient (maximum of 5 data points per patient).

#### Mortality

12-month mortality rates were assessed by country and distinguishing between patients with active cancer and without active cancer. Additionally, the observed mortality rates were compared with the age- and gender-matched mortality rate from the UK general population (year 2013) [22]. The association between mortality and baseline characteristics (the same as those in the QOL analysis plus PE symptoms and EQ-5D-5L index score at baseline) was examined. In addition to the evaluation of the total patient population, a distinction was made between patients with or without active cancer. Lacking exact death dates, logistic regression with backward stepwise elimination was used to analyze the mortality data instead of Cox-regression. In logistic regression models, all baseline characteristics were considered and selected by backward stepwise elimination. Furthermore, the regression was carried out with and without health-related quality of life at baseline (the presenting EQ-5D-5L index scores) as a predictor because quality of life may affect mortality but that also a high probability of mortality may affect quality of life. In these analyses including EQ-5D-5L index scores, PE symptoms were excluded because of the correlation between PE symptoms and baseline EQ-5D-5L index score. Analyses of country differences were addressed in an additional analysis.

### Analyses and statistics

Descriptive statistics of baseline measurements were provided, including demographics, clinical factors (previous VTE event, PE with DVT and provoked^1^), previous clinical events (within 3 years prior to enrollment: myocardial infarction, coronary heart disease, percutaneous coronary intervention, atrial fibrillation, transient ischemic attack, stroke, and bleeding event), risk factors (within the past 3 months or ongoing: active cancer, prolonged immobilization (> 5 days in bed), varicose veins, history of major surgery or trauma), comorbidities (hypertension, congestive heart failure, vascular disease, dyslipidemia, diabetes, chronic venous insufficiency, renal disease, liver disease, chronic respiratory disease, arthritis, bone fracture/soft tissue trauma, lower extremity paralysis, alcohol use, smoking history, thrombophilia, and cardiovascular disease), and the presence of PE symptoms. Treatment at baseline, i.e., the use of heparin, vitamin K antagonists (VKA), or direct oral anticoagulants (DOACs), was tabulated. Patients receiving ongoing treatment for cancer were labeled as “active cancer” patients. Country-specific differences were analyzed in which Austria, Switzerland, and Germany were combined into one pre-specified region label (DACH). The DACH countries that were grouped in a cluster as patient population, practice patterns, and healthcare systems were assumed to be similar and the numbers of sites for Germany, Austria, and Switzerland were, respectively, 74, 5, and 3. Detailed clinical variables can be found elsewhere [19].

#### Missing data and sensitivity analysis

There was missing information (loss to follow-up, death, or incomplete data). No imputation was conducted for any missing value in the above main analysis. However, the difference in baseline characteristics between patients who completed the follow-ups and those who did not were tested, using *χ*^2^, rank sum test, or *t* test when appropriate. Furthermore, a sensitivity analysis, using the multiple imputation technique to generate values for missing data, was conducted to evaluate the potential impact of missing data. Following the technique of chained equations, 20 imputation datasets were generated. [23] Baseline characteristics and available index scores at different time points were used for imputing missing data.

## Results

### Patient characteristics

The registry enrolled 1399 PE patients, and baseline characteristics are listed in Table 1. 25.2% of patients were from France, followed by Italy 23.7%, Spain 23.4%, DACH 17.2%, and UK 10.5%. The most common comorbidity was hypertension (46.3%). In 8.6% of patients, active cancer was diagnosed. The most common reported PE symptoms included dyspnea and chest pain, 75.6% and 45.5%, respectively. The majority of patients received heparin at baseline (85.4%). VKA was prescribed for 57.4% of patients and 21.2% used NOACs (mostly used in patients in France and DACH, 32.6% and 55.2%, respectively). The detailed baseline characteristics of the country subgroup can be found elsewhere [15].

**Table 1:** Patients’ characteristics at baseline

Baseline, %	Total *N* = 1399	Active cancer		Baseline, %	Total *N* = 1399	Active cancer	
		No, *N* = 1279	Yes, *N* = 120			No, *N* = 1279	Yes, *N* = 120
Age, years, mean (SD)	62.3 (17.1)	61.8 (17.4)	67.2 (11.5)*	Comorbidities			
Male	53.2	52.4	61.7	Active cancer	8.6	0	100
BMI, mean (SD)	28.16 (6.0)	28.4 (5.9)	25.7 (5.5)*	Hypertension	46.3	46.6	43.3
Highest graduation				Congestive heart failure	5.9	5.9	6.7
Primary school	32.0	31.7	35	Vascular disease	7.1	6.8	10*
Secondary school	41.8	41.9	40.8	Dyslipidemia	26.4	26.2	28.3
Above	21.9	22.1	20	Diabetes	11.2	10.8	15
Marital status				Chronic venous insufficiency	14.2	14.5	10.8
Single	14.2	16.0	9.2	Renal disease	6.4	6.7	4.2
Married/living as married	62.2	61.2	72.5	Liver disease	2.6	2.0	9.2*
Separated/divorced	5.3	5.7	0.8	Chronic respiratory disease	10.7	10.3	15
Widowed	14.4	14.4	15	Arthritis	6.0	6.3	2.5
Other	1.2	1.3	0	Bone fracture/soft tissue trauma	10.0	10.3	5.8
Country				Lower extremity paralysis	1.1	1.2	0
France	25.2	25.5	21.7	Thrombophilia	5.1	5.5	0.8*
DACH	17.2	18.3	5.8	Cardiovascular disease	16.8	16.7	18.3
Italy	23.7	22.7	35*	Alcohol use	15.6	16.3	7.5*
Spain	23.4	22.4	34.2*	Smoking history	33.1	32.0	45.8*
UK	10.5	11.2	3.3	Risk factors (within past 3 month or ongoing)			
Previous clinical event (within 3 yr. prior to enroll.)				Prolonged immobilization	17.8	17.9	16.7
Myocardial infarction	3.7	3.8	3.3	>5 day in bed	11.8	11.5	15
Coronary artery disease	3.8	3.8	3.3	Varicose veins	17.5	18.2	10
Percutaneous coronary intervention	2.4	2.4	2.5	Major surg. or trauma	14.0	13.9	15
Atrial fibrillation	4.7	4.5	7.5	PE symptoms present			
Transient ischemic attack	2.65	2.66	2.5	Dyspnea	75.6	76.2	70*
Stoke	2.7	2.8	1.7	Chest pain	45.5	47.2	28.3*
Bleeding event	4.2	4.0	6.7	Cough	16.8	16.7	18.3
Clinical factors				Hemoptysis	3.4	3.6	1.7
Previous VTE event	20.2	20.9	13.3	Syncope	8.2	8.3	6.7
With concomitant DVT	46.5	45.5	57.5*	Palpitations	7.9	7.7	9.2
Provoked	27.5	27.3	30	Fever	7.8	7.9	6.7
Baseline treatment				Cyanosis	2.2	2.1	3.3
Use of heparin	85.4	85.4	84.9	Tachypnea	16.2	16.0	17.5
Use of VKA	57.4	61.5	13.5*	Tachycardia	16.7	16.4	19.2
Use of NOACs	21.2	22.7	5.9*	Cardiogenic shock	1.5	1.4	2.5
				Others	7.6	8.0	3.3

*DACH* Austria, Switzerland, and Germany; *VTE* venous thromboembolism; *DVT* deep vein thrombosis; *VKA* vitamin K antagonists; *NOAC* non-VKA oral anticoagulants

*Difference between the groups with/without active cancer reached statically significant level *p *< 0.05

Patients with active cancer were significantly older, and had lower BMI and the PE was more often diagnosed with a DVT. Patients without active cancer reported dyspnea and chest pain more often.

The percentage of active cancer patients differed substantially between countries. In DACH and the UK, less than 3% of the included patients had active cancer, in France 7%, and in Italy and Spain more than 12% of the patients that were included had active cancer. At baseline, patients with active cancer were most often treated with heparin and significantly less (than patients without active cancer) with VKAs or NOACs.

### Missing data

It was found that patients who did not complete EQ-5D-5L at 12-month follow-up were younger, mostly British, with more alcohol/smoking history and active cancer, but less PE with DVT, hypertension, dyslipidemia, and chronic venous insufficiency. Patients with missing data at other follow-ups were also younger, British, with more smoking history and active cancer, but less dyslipidemia and chronic venous insufficiency.

### Health-related quality of life

**Fig. 1:**
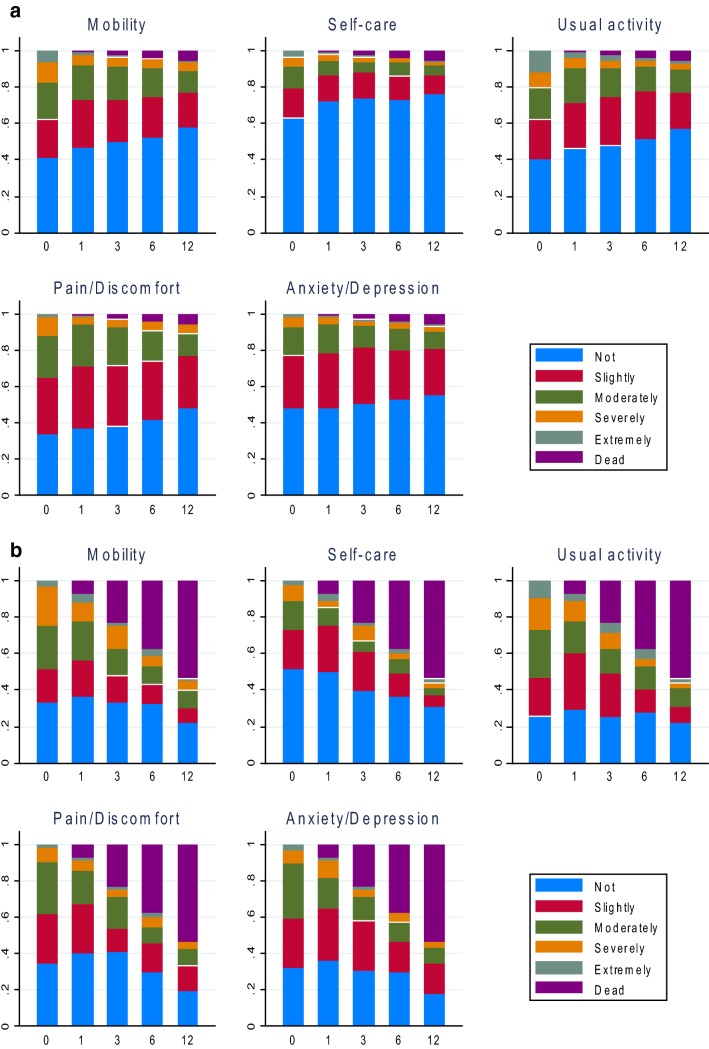
**a** Distribution of EQ-5D-5L domains and death at baseline and follow-up: patients without active cancer. **b**. Distribution of EQ-5D-5L domains and death at baseline and follow-up: patients with active cancer

At baseline, 1324 patients filled in EQ-5D-5L, 897 at 1 month, 809 at 3 months, 748 at 6 months, and 634 at 12 months. Figure 1 presents the distribution of each dimension of EQ-5D-5L and death for patients without active cancer (a) and those with active cancer (b). With respect to patients without active cancer, all dimensions clearly demonstrated a similar trend with patients reporting fewer problems or indicating lower severity of problems over time, while the number of deaths increased over time. Findings were similar for patients with active cancer, less obvious in Fig. 1b due to the high mortality rate. For the whole cohort, baseline EQ-5D-5L index score was the lowest (0.712 [SD 0.265]), and gradually improved to 0.788 (0.212), 0.805 (0.209), and 0.817 (0.213) at 1-, 3-, and 6-month follow-up, respectively, and finally reached 0.835 (0.212) at 12-month follow-up. When death was scored as zero, the average index score at each follow-up was 0.712 (0.265), 0.774 (0.234), 0.768 (0.265), and 0.743 (0.330) at 1, 3, 6, and 12 months, respectively. The norm value index score for an age/gender-matched general population is 0.838.

**Fig. 2:**
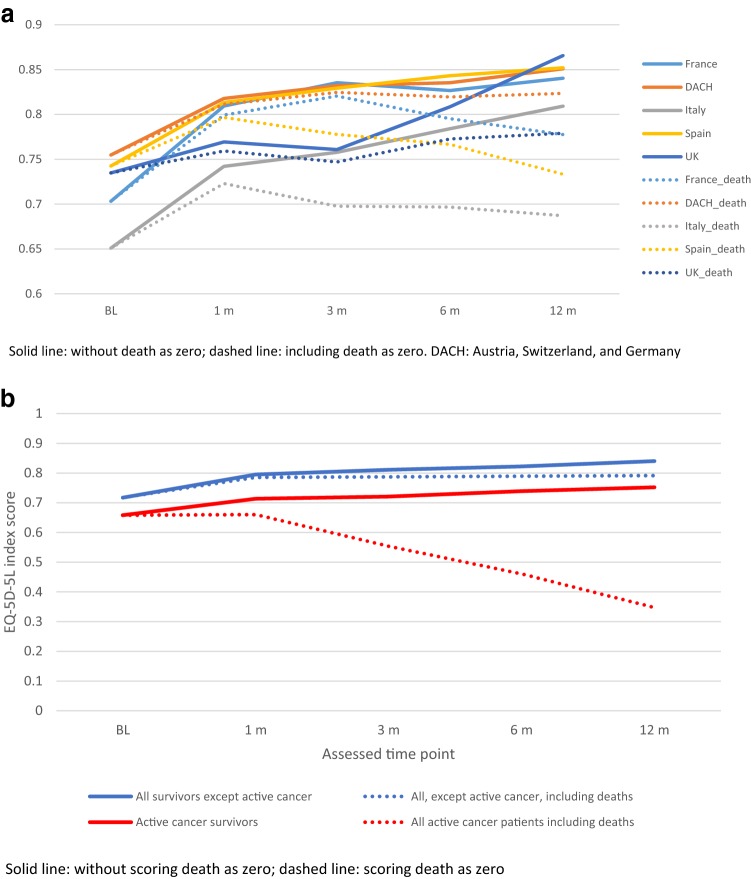
EQ-5D-5L index score at each follow-up, by a. country and b. cancer subgroup. **a** By country. Solid line: without death as zero; dashed line: including death as zero. DACH: Austria, Switzerland, and Germany. **b** By active cancer subgroup. Solid line: without scoring death as zero; dashed line: scoring death as zero

Figure 2 presents the index scores with/without death as zero over time by (a) country and (b) cancer subgroups. Over time, France, DACH, and Spain had similar index scores, whereas Italy consistently had lower scores (Fig. 2a). The difference in index scores between cancer subgroups remained stable over time (solid lines in Fig. 2b). However, when death was scored as zero, the gap between two groups increased substantially as a higher proportion of death occurred in the cancer group and subsequently lowered the average of index score (dashed lines).

The results from the stepwise regression on EQ-5D-5L index scores for patients without active cancer—excluding the presence of bleeding events and VTE events—showed significant negative effects of a number of factors, including increasing age, being female, increasing BMI, previous stroke, prolonged immobilization, > 5 days in bed, with comorbidities of vascular disease, diabetes mellitus, chronic respiratory disease, arthritis, and bone fracture/soft tissue trauma (Table 2). However, having varicose veins or dyslipidemia was associated with a higher index score. When including VTE events and bleeding events into the model, the results showed that only bleeding and not VTE events were found to have a significant effect on quality of life. For patients with active cancer, only age and previous stroke were significant predictors, while VTE/bleeding events had no impact. Furthermore, no country effects were encountered after including country-specific variables in both groups.

**Table 2:** Analysis of EQ-5D-5L index score, Tobit model with repeated measures results

	Without VTE/bleeding events (*n* = 1191, Obs = 4024)					With VTE/bleeding events (*n* = 1191, Obs = 3875)				
	Coef.	Std. Err.	*p* > z	[95% Conf.	Interval]	Coef.	Std. Err.	*p* > z	[95% Conf.	Interval]
Patients without ACTIVE cancer										
Constant	0.974	0.044	< 0.001	0.888	1.060	0.970	0.044	< 0.001	0.884	1.057
Age	− 0.001	0.000	0.006	− 0.002	0.000	− 0.001	0.000	0.011	− 0.002	0.000
Male	0.105	0.013	< 0.001	0.079	0.131	0.105	0.013	< 0.001	0.079	0.131
Body mass index	− 0.004	0.001	0.001	− 0.006	− 0.002	− 0.004	0.001	0.001	− 0.006	− 0.002
Previous stroke	− 0.115	0.040	0.004	− 0.192	− 0.037	− 0.120	0.040	0.003	− 0.199	− 0.042
Prolong immobilization	− 0.074	0.021	< 0.001	− 0.114	− 0.034	− 0.072	0.021	0.001	− 0.112	− 0.031
> 5 day in bed	− 0.071	0.024	0.003	− 0.118	− 0.023	− 0.068	0.024	0.005	− 0.116	− 0.021
Varicose veins	0.036	0.017	0.037	0.002	0.070	0.038	0.017	0.03	0.004	0.072
Vascular disease	− 0.092	0.027	0.001	− 0.145	− 0.039	− 0.090	0.027	0.001	− 0.143	− 0.036
Dyslipidemia	0.040	0.015	0.010	0.010	0.070	0.038	0.015	0.014	0.008	0.068
Diabetes mellitus	− 0.059	0.022	0.007	− 0.101	− 0.016	− 0.055	0.022	0.011	− 0.098	− 0.013
Chronic respiratory disease	− 0.057	0.022	0.009	− 0.100	− 0.015	− 0.059	0.022	0.007	− 0.102	− 0.016
Arthritis	− 0.068	0.026	0.008	− 0.119	− 0.018	− 0.065	0.026	0.013	− 0.116	− 0.014
Bone fracture/soft tissue trauma	− 0.073	0.022	0.001	− 0.116	− 0.030	− 0.074	0.022	0.001	− 0.117	− 0.031
VTE event during follow-up	Not included					− 0.061	0.043	0.158	− 0.145	0.024
Bleeding event during follow-up	Not included					− 0.050	0.022	0.027	− 0.094	− 0.006

	Without VTE/bleeding events (*n* = 110, Obs = 321)					With VTE/bleeding events (*n* = 110, Obs = 296)				
	Coef.	Std. Err.	*p* > z	[95% Conf.	Interval]	Coef.	Std. Err.	*p* > z	[95% Conf.	Interval]
Patients with ACTIVE cancer										
Constant	1.236	0.161	< 0.001	0.921	1.552	1.237	0.165	0 < 0.001	0.914	1.560
Age	− 0.008	0.002	0.001	− 0.012	− 0.003	− 0.008	0.002	0.001	− 0.013	− 0.003
Previous stroke	− 0.762	0.313	0.015	− 1.376	− 0.148	− 0.758	0.318	0.017	− 1.382	− 0.134
VTE event during follow-up	Not included					0.069	0.182	0.704	− 0.288	0.427
Bleeding event during follow-up	Not included					− 0.076	0.091	0.400	− 0.254	0.102

*N* number of patients, *Obs*. number of observations of index scores (per patient could contribute multiple, up to 5, observations), *VTE* venous thromboembolism

The full list of covariate set includes age, gender, BMI, clinical factors (previous VTE event, PE with DVT), previous clinical events (within 3 years prior to enrollment: myocardial infarction, coronary heart disease, percutaneous coronary intervention, atrial fibrillation, transient ischemic attack, stroke, and bleeding event), risk factors (within the past 3 months or ongoing: prolonged immobilization (> 5 days in bed), varicose veins, history of major surgery or trauma), comorbidities (hypertension, congestive heart failure, vascular disease, dyslipidemia, diabetes, chronic venous insufficiency, renal disease, liver disease, chronic respiratory disease, arthritis, bone fracture/soft tissue trauma, lower extremity paralysis, alcohol use, smoking history, and thrombophilia), and VTE/bleeding events during follow-up

The results of sensitivity analysis, addressing potential selection bias, were mostly in line with the main results above. As shown in Appendix Fig. 3, after the imputation, the trend of improvement over time was less obvious compared to that in the main analysis (without imputation, Fig. 2b). The same set of significant covariates were observed in the Tobit regression using imputed data.

**Fig. 3:**
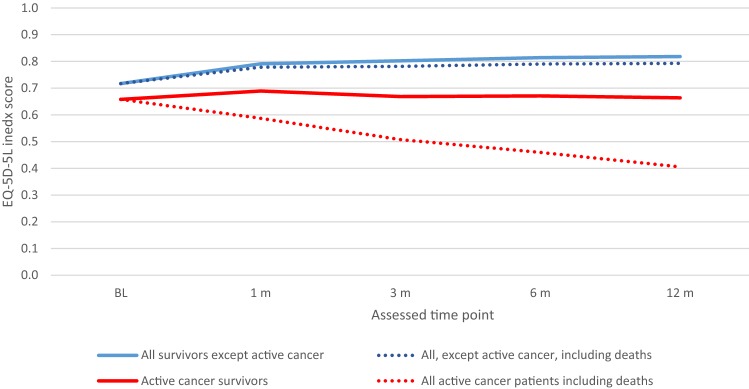
Imputed EQ-5D-5L index score at each follow-up, by cancer subgroup

### Mortality

In total, 8.1% of patients (102 out of total 1263 patients with mortality information) died during the 12-month period. Main outcome mortality rate at 12 months differed among countries: 5.2% (17/326) in France, 1.4% (3/212) in DACH, 16.8% (52/310) in Italy, 9.1% (27/298) in Spain, and 2.6% (3/117) in the UK (*p* value ≤ 0.0001). The mortality rate was significantly higher in patients with active cancer than in those without active cancer (42.7% vs. 4.7%, *p* value ≤ 0.0001). In comparison, the age- and gender-adjusted (reflecting the total PE study population) mortality rate of a UK general population in 2013 is around 2.5%. Table 3 presents the cause of death at each follow-up time point of total PE patients and by cancer subgroups. Among all deaths, only 6.9% were VTE-related—highest in patients without active cancer.

**Table 3:** Mortality rate by each follow-up and cancer subgroup

PE	Baseline	Month 1	Month 3	Month 6	Month 12
All					
Total	1398	1388	1327	1307	1263
Death, *n*(%)	14 (1.00)	34 (2.45)	59 (4.45)	79 (6.04)	102 (8.08)
Reason for death					
Missing/unknown	–	2 (5.9)	2 (3.4)	4 (5.1)	6 (5.9)
VTE-related death	5 (35.7)	6 (17.6)	7 (11.9)	7 (8.9)	7 (6.9)
Cardiovascular death	3 (21.4)	5 (14.7)	9 (15.3)	12 (15.2)	18 (17.6)
Other	6 (42.9)	21 (61.8)	41 (69.5)	56 (70.9)	71 (69.6)
Without active cancer					
Total	1278	1270	1213	1194	1151
Death, *n*(%)	11 (0.86)	23 (1.81)	38 (3.13)	45 (3.8)	54 (4.69)
Reason for death					
Missing/unknown	–	1 (4.3)	1 (2.6)	3 (6.7)	2 (3.7)
VTE-related death	5 (45.5)	6 (26.1)	6 (15.8)	6 (13.3)	6 (11.1)
Cardiovascular death	2 (18.2)	4 (17.4)	8 (21.1)	11 (24.4)	13 (24.1)
Other	4 (36.4)	12 (52.2)	23 (60.5)	25 (55.6)	30 (55.6)
With active cancer					
Total	120	118	114	113	112
Death, *n*(%)	3 (2.5)	11 (9.32)	21 (18.4)	34 (30.1)	48 (42.9)
Reason for death					
Missing/unknown	–	1 (9.1)	1 (4.8)	1 (2.9)	4 (8.3)
VTE-related death	–	–	1 (4.8)	1 (2.9)	1 (2.1)
Cardiovascular death	1 (33.3)	1 (9.1)	1 (4.8)	1 (2.9)	5 (10.4)
Other	2 (66.7)	9 (81.8)	18 (85.7)	31 (91.2)	41 (85.4)

*VTE* venous thromboembolism

Table 4 summarizes the effect of significant baseline characteristics on mortality after backward elimination for the total patient population, and with a breakdown in patients with active cancer and those without, including and excluding baseline EQ-5D-5L index score as an additional explanatory variable. In patients without active cancer, increasing age, low BMI, having prolonged immobilization, vascular disease, diabetes mellitus, and cardiogenic shock at the time of PE diagnosis showed a significant association with higher all-cause mortality. In active cancer patients, a history of smoking and tachycardia were found to be significant predictors. When the baseline EQ-5D-5L index score was entered into the models, quality of life showed a significant association with mortality in both active and non-active cancer patients without major changes in the effects of the other variables.

**Table 4:** Analysis of mortality, logistic regression results adjusted for significant baseline variables

	Baseline characteristics (*n* = 1183)					Baseline characteristics (excluding PE Symptoms^a^) and EQ-5D-5L index score (*n* = 1198)				
	Odds ratio	Std. err.	*p* value	[95% conf.	Interval]	Odds ratio	Std. err.	*p* value	[95% conf.	Interval]
Total PE cohort										
Constant	0.017	0.018	< 0.001	0.002	0.135	0.201	0.231	0.162	0.021	1.912
Age	1.046	0.011	< 0.001	1.025	1.068	1.043	0.011	< 0.001	1.022	1.065
BMI	0.905	0.025	< 0.001	0.857	0.955	0.887	0.026	< 0.001	0.837	0.939
Active cancer	13.137	3.600	< 0.001	7.678	22.478	12.348	3.495	< 0.001	7.090	21.504
Prolong immobilization	2.587	0.777	0.002	1.436	4.661					
Vascular disease	2.361	0.789	0.010	1.226	4.545					
Previous AF	2.531	1.007	0.020	1.16	5.52	2.871	1.159	0.009	1.301	6.334
Smoking history	1.931	0.495	0.010	1.168	3.191	1.967	0.534	0.013	1.155	3.349
Palpitations	2.276	0.874	0.032	1.072	4.831	Not included^a^				
EQ-5D-5L index score at baseline	Not included					0.095	0.040	< 0.001		

	Baseline characteristics (*n* = 1071)					Baseline characteristics (excluding PE Symptoms^a^) and EQ-5D-5L index score (*n* = 1090)				
	Odds ratio	Std. err.	*p* value	[95% conf.	Interval]	Odds ratio	Std. err.	*p* value	[95% conf.	Interval]
Patients without ACTIVE cancer										
Constant	0.194	0.280	0.257	0.011	3.294	0.502	0.775	0.655	0.024	10.315
Age	1.058	0.014	< 0.001	1.030	1.086	1.054	0.014	< 0.001	1.027	1.082
Body mass index	0.796	0.035	< 0.001	0.730	0.869	0.808	0.035	< 0.001	0.742	0.881
Prolong immobilization	2.570	0.866	0.005	1.328	4.975	2.053	0.737	0.045	1.016	4.148
Vascular disease	3.580	1.353	0.001	1.707	7.510	2.493	1.014	0.025	1.124	5.531
Diabetes mellitus	2.575	0.985	0.013	1.217	5.448	2.534	0.996	0.018	1.173	5.474
Cardiogenic shock	17.573	11.213	< 0.001	5.032	61.373	Not included^a^				
EQ-5D-5L index score at baseline	Not included					0.220	0.117	0.004	0.078	0.622

	Baseline characteristics (*n* = 112)					Baseline characteristics (excluding PE Symptoms^a^) and EQ-5D-5L index score (*n* = 108)				
	Odds ratio	Std. err.	*p* value	[95% conf.	Interval]	Odds ratio	Std. err.	*p* value	[95% conf.	Interval]
Patients with ACTIVE cancer										
Constant	0.311	0.101	< 0.001	0.164	0.588	3.232	1.934	0.050	1.001	10.440
Smoking history	2.964	1.246	0.010	1.300	6.758	3.346	1.514	0.008	1.379	8.122
Tachycardia	6.295	3.633	0.001	2.031	19.510	Not included^a^				
EQ-5D-5L index score at baseline	Not included					0.035	0.031	< 0.001	0.006	0.193

The full list of covariate set includes age, gender, BMI, clinical factors (previous VTE event, PE with DVT), previous clinical events (within 3 years prior to enrollment: myocardial infarction, coronary heart disease, percutaneous coronary intervention, atrial fibrillation, transient ischemic attack, stroke, and bleeding event), risk factors (within the past 3 months or ongoing: active cancer, prolonged immobilization (> 5 days in bed), varicose veins, history of major surgery or trauma), comorbidities (hypertension, congestive heart failure, vascular disease, dyslipidemia, diabetes, chronic venous insufficiency, renal disease, liver disease, chronic respiratory disease, arthritis, bone fracture/soft tissue trauma, lower extremity paralysis, alcohol use, smoking history, and thrombophilia), the presence of PE symptoms, and EQ-5D-5L index score at baseline

*PE* pulmonary embolism

^a^Baseline PE symptoms were not considered in this model (i.e., Dyspnea, Chest pain, Cough, Hemoptysis, Syncope, Palpitations, Fever, Cyanosis, Tachypnea, Tachycardia, Cardiogenic shock, Others.)

After repeating the above regression analyses with the addition of “country” as a predictor (UK as the reference country), France and DACH had significantly lower mortality rates in patients without active cancer (OR 0.243 and 0.293 for France and DACH, respectively). When including quality of life as a predictor, only Italy showed a significant association with decreased survival compared to the UK (OR 0.3023). By contrast, no country effect was found in patients with active cancer.

## Discussion

PE patients had worse EQ-5D-5L index scores compared to a population norm and the scores gradually improved among survivors during the follow-up, with the average returning close to the norm. Age and previous stroke were independently associated with low index scores in both groups with/without active cancer. Furthermore, it was found that bleeding, but not recurrences, had an impact on HrQoL of patients without active cancer. The study also shows that the average mortality rate at 12 months was 8.1%, varying between countries, and was significantly different between patients with active cancer and those without. Significant predictors of mortality included increasing age, low BMI, prolonged immobilization, vascular disease, diabetes mellitus, and presenting with cardiogenic shock for patients without active cancer; smoking history and tachycardia for patients with active cancer.

### Utility

Our finding was aligned with previous studies that reported the HrQoL scores of PE patients were lower than those of a general population [10, 12, 24]. The reported EQ-5D-3L index scores were 0.75 for female and 0.85 for male PE patients [10], and 0.68 and 0.82 for VTE patients at baseline (newly diagnosed) and 3-month follow-up, respectively [25]. However, it should be noted that these scores are not directly comparable mainly as our study used EQ-5D-5L version. The average index score for PE patients at baseline was similar to patients who were awaiting renal transplantation (0.773) [26]. Although the index scores gradually improved among PE survivors during the follow-up, the average was still lower than the norm (age and gender adjusted). Of note, the observed improvement over time was driven by the surviving patient population, and with the inclusion of patients who died, the average index score decreased. Patients who dropped out of the study tended to be less healthy and have lower index scores. Thus, the trend observed should be interpreted with caution. As demonstrated in Appendix Fig. 3, after including the imputed missing values, the improvement over time was minimized.

Among PE patients without active cancer, the effects of bleeding were significantly associated with a lower quality of life as measured by the utility score, but not the effects of recurrent VTE. This might be explained by bleeding being associated with high morbidity and higher case-fatality than recurrent VTE [27]. It is possible that there was a lack of power to detect changes relating to recurrent VTE as around 1% of patients experienced recurrences at each follow-up. This association was not observed in the patients with active cancer.

We also found that a history of varicose veins and dyslipidemia were significantly associated with better quality of life, which may be due to chance, unobserved interaction, or confounding or possibly related to the patients’ interest in general health and preventive therapies.

None of the countries were a significant predictor of HrQoL. However mortality, after correcting for baseline characteristics and events, showed significant differences between countries. Marvig et al. [25] reported the between-country difference in HrQoL of VTE patients and suggested various factors that might explain the variation, such as different response style, reference levels, external factors, and especially cultural differences. In contrast, across countries in our study the different case mix might be the main factor explaining the data variation, since country data were a non-significant factor predicting the index score after controlling for case mix. The lower index score observed in Italy (as shown in Fig. 2a) is likely to be explained by the severity of cases. More specifically, Italian and Spanish patients were recruited from the hospital setting only and had more active cancer patients, whereas in France, DACH, and the UK both inpatients and outpatients were included. Detailed patient characteristics can be found in the accompanying paper [15].

### Mortality

The mortality rate at one month was 2.4% and at 12 months was 8.1%. This was low compared to the 30-day all-cause mortality rate of 5.9% reported in the RIETE registry [14] and 5.5% reported in the EMPEROR registry [28]. This might be explained by the design of the PREFER registry in that patients were recruited in the post-acute treatment period, up to two weeks from the occurrence of the index event and were to be followed for 12 months. Therefore, patients were survivors of the initial period and likely to be healthier. In addition, a wide range of 1-year mortality rates was observed across countries (from 1.4% in DACH to 16.8% in Italy). Several factors might have contributed to this variation. Not all countries included the same “mix” of patient comorbidities and there was variation in the inpatient and outpatient disease management across countries.

The mortality regression results also reflected the observed heterogeneity between countries. For instance, among patients without active cancer, after controlling for case mix (age, gender, BMI, previous clinical events, clinical factors, comorbidities, risk factors and PE symptoms at baseline, as well as EQ-5D-5L index score) the survival of patients in DACH and France was better than those in the UK and Spain, and those in Italy had a worse prognosis. This implies that there may still be other factors (residual confounding) that contribute to this difference in mortality rates in Italy and Spain, which were not included in this analysis. Moreover, one might argue that the term ‘country’ effect here should be interpreted with caution since PE patients recruited in the PREFER study in each country might not reflect an average PE patient in that country.

This study also showed a significantly higher mortality rate in patients with active cancer compared with those without. It is generally accepted that 15–20% of all PE patients have active cancer at the time of PE diagnosis [29], while the proportion of PE patients with active cancer was 8.6% in our study. Hence in the PREFER registry in VTE, cancer patients were less likely to be included. As such, the current mortality estimate can be considered conservative.

It has been reported that the PE associated mortality rate has reduced significantly over time. In the study by Jiménez and his colleagues, based on a large registry, risk-adjusted rates of 30-day all-cause mortality decreased from 6.6% during 2001–2005 to 4.9% during 2010–2013; rates of 30-day PE-related mortality decreased from 3.3 to 1.8% [14]. Their study also reported accompanying changes in the length of hospital stay (shorter over the years) and changes in the initial treatment in the same period.

### Strengths and limitations

The PREFER in VTE registry provides a rich data source of epidemiology, management, and outcomes of VTE based on a large registry sample with frequent measurements in a real-world setting. It collected data from seven European countries, and provides a very much needed input for overcoming the scarcity of PE evidence originating from the region. Furthermore, the design of PREFER—without exclusion criteria—allowed all type of PE patients to be recruited, providing a source of heterogeneous data in a real-world setting. The registry was conducted at time when NOAC availability varied among countries, offering a unique opportunity for understanding the potential uptake of NOACs.

Weaknesses include missing data and selection bias for healthier patients. Missing data are a common problem with real-world evidence. There are many possible approaches to impute missing values; however, in this study the multiple imputation method was employed and resulted in a similar finding as that of the main analysis, particularly with respect to the factors determining the HrQoL. Registries are less selective than clinical trials but still select healthier and more compliant patients. Furthermore, the absence of any information on cancer types prevents detailed analyses in understanding any possible interaction between the type of cancer and PE. That the causes of death were not adjudicated by a centralized ad hoc committee is also a limitation. A further limitation is the multiplicity of the statistical analysis leading to possible chance findings. Finally, due to the design of the registry, the generalizability of country-specific data might be limited.

## Conclusion

Reductions in health-related quality of life following pulmonary embolism and treatment improved over time among survivors. The most concerning complication of anticoagulation, bleeding (but not recurrences), had an association with worse health-related quality of life. This should impact the assessment of risk benefit in the era of NOAC therapy, as NOACs result in less bleeding compared with VKA. In the post-acute treatment setting, mortality rates remained high and were strongly related to the presence of active cancer. There also seem to be some country differences in survival following pulmonary embolism. The current study may guide further data collection and enhance discussion concerning the management decisions in order to optimize outcomes for PE patients in Europe.

## Appendix

See Fig. 3.

## Footnotes

1 Provoked PE was defined as having prolonged immobilization, > 5 days in bed, or history of major surgery or trauma.
